# The Role of Artificial Intelligence in Developing a Future‐Ready Nursing Workforce: A Systematic Review

**DOI:** 10.1155/jonm/8272597

**Published:** 2026-11-01

**Authors:** Abu Zafor Md Shaleah, Rajib Kumar Biswas, Moustaq Karim Khan Rony, Most. Tahmina Khatun, Salma Akhter, Most Baby Najnin, Sujoy Biswas, Maryam Alharrasi

**Affiliations:** ^1^ School of Graduate Studies Rutgers University Newark New Jersey USA rutgers.edu; ^2^ Nell Hodgson Woodruff School of Nursing Emory University Atlanta USA emory.edu; ^3^ Miyan Research Institute International University of Business Agriculture and Technology Dhaka Bangladesh iubat.edu; ^4^ Faculty of Graduate Studies University of Kelaniya Colombo Sri Lanka kln.ac.lk; ^5^ Department of Nursing West Najran Hospital for Maternity and Children Najran Saudi Arabia; ^6^ Graduate School of Biomedical and Health Science Hiroshima University Hiroshima Japan hiroshima-u.ac.jp; ^7^ Nursing Service Dhaka Medical College Hospital Dhaka Bangladesh dmc.edu.bd; ^8^ College of Nursing Sultan Qaboos University Muscat Oman squ.edu.om

**Keywords:** artificial intelligence, clinical decision support, nursing education, nursing workforce, workforce readiness

## Abstract

**Background:**

Artificial intelligence (AI) is increasingly transforming health care through advanced technologies that support clinical practice, education, workforce management, and decision making. As healthcare systems face growing challenges related to workforce shortages, increasing service demands, and rapid digital transformation, there is a need to understand how AI can contribute to the development of a future‐ready nursing workforce equipped with the competencies required for modern healthcare environments.

**Aim:**

This systematic review aimed to examine the role of AI in developing a future‐ready nursing workforce and to synthesize current evidence regarding its applications, benefits, challenges, and implications for workforce preparedness.

**Methods:**

A systematic review was conducted in accordance with PRISMA 2020 and was registered in PROSPERO. PubMed, Scopus, Web of Science, CINAHL, and IEEE Xplore were searched for studies published between January 2015 and February 2026. Eligible primary studies examined an identifiable AI technology or an AI‐specific construct in nursing education, clinical practice, workforce development, competency development, workforce planning, or professional preparedness. Methodological quality was assessed using the Mixed Methods Appraisal Tool, and findings were synthesized narratively.

**Results:**

A total of 38 primary empirical studies satisfied the AI‐specific eligibility criteria. The evidence was concentrated in cross‐sectional (*n* = 15) and qualitative (*n* = 11) designs, with fewer development/prototype (*n* = 6), mixed methods (*n* = 3), randomized or quasi‐experimental (*n* = 2), and methodological (*n* = 1) studies. AI‐supported education, clinical decision support, workforce forecasting, and competency development showed promising benefits, but much of the evidence was based on perceptions, simulations, prototypes, or single‐site evaluations rather than long‐term implementation. Recurrent barriers included limited AI literacy, ethical and privacy concerns, algorithmic bias, infrastructure constraints, workflow integration, and organizational readiness.

**Conclusion:**

AI has promising, but still uneven, potential to support a future‐ready nursing workforce. Current evidence supports cautious and staged integration rather than uncritical scale‐up. Standardized AI and digital competency frameworks, human oversight, governance, infrastructure readiness, and prospective evaluation of effectiveness, equity, and cost are needed before widespread deployment.

**Implications for Nursing Management:**

Nursing managers should prioritize standardized AI and digital competency development, readiness assessment, pilot testing, and governance before organization‐wide implementation. Decisions about AI adoption should also consider infrastructure, training, workflow redesign, total cost of ownership, and sustainability, particularly in resource‐constrained settings.

## 1. Introduction

Artificial intelligence (AI) has emerged as one of the most influential technological innovations shaping modern healthcare systems [1]. Through advanced computational techniques such as machine learning, natural language processing, predictive analytics, and intelligent automation, AI is transforming the way healthcare services are delivered, managed, and evaluated [2, 3]. Healthcare organizations worldwide are increasingly adopting AI‐driven solutions to improve efficiency, support clinical processes, enhance patient outcomes, and optimize resource utilization [4]. At the same time, healthcare systems face growing pressures associated with aging populations, increasing prevalence of chronic diseases, workforce shortages, rising healthcare expenditures, and expanding demands for high‐quality care [5, 6]. These challenges have intensified the need for innovative approaches capable of strengthening healthcare capacity while maintaining safe and effective service delivery [7]. Within this rapidly evolving environment, nursing professionals occupy a central position in patient care and healthcare operations, making the relationship between AI and nursing workforce development an area of increasing importance [8]. Understanding how AI can support the preparation, sustainability, and advancement of the nursing workforce is therefore essential for future healthcare planning [9].

The concept of a future‐ready nursing workforce extends beyond traditional clinical competence and encompasses adaptability, technological proficiency, digital literacy, critical thinking, and the ability to function effectively within increasingly complex healthcare environments [10]. Contemporary nursing practice requires professionals to engage with electronic health records, digital communication systems, telehealth platforms, health informatics applications, and data‐driven decision‐making processes [11]. As technological integration continues to expand across healthcare settings, nurses are expected to develop competencies that enable them to work alongside emerging technologies while maintaining patient‐centered care [12, 13]. Educational institutions, healthcare organizations, and policymakers have recognized the need to prepare nurses for these evolving responsibilities [14]. Consequently, attention has shifted toward identifying strategies that can enhance workforce readiness and support professional development in an era characterized by rapid technological transformation [15]. AI has gained significant attention in this context because of its potential to facilitate learning, strengthen workforce capabilities, and support the acquisition of competencies necessary for modern healthcare practice [16].

Recent years have witnessed a growing body of research exploring AI across nursing education, clinical practice, workforce management, professional development, healthcare administration, and quality improvement [17–21]. AI has also generated interest as a possible response to staffing pressures, workload demands, and continuing professional upskilling [22]. Despite this expansion, the evidence remains dispersed across application areas and study designs [23, 24]. More broadly, nursing workforce sustainability and educational reform are shaped by policy, organizational, and academic‐clinical factors that differ across health systems [25, 26]. Within AI‐focused nursing research, heterogeneity in technologies, implementation settings, evaluation methods, and outcomes complicates direct comparison and interpretation [15, 16, 23].

Given the accelerating integration of AI into healthcare systems and the growing emphasis on workforce preparedness, a comprehensive synthesis of current evidence is needed to inform education, practice, leadership, and policy. Evaluating the role of AI in developing a future‐ready nursing workforce can provide valuable insights into emerging opportunities, implementation considerations, and areas requiring further investigation. Such knowledge is important for guiding investments in workforce development, informing curriculum design, supporting organizational planning, and promoting evidence‐based adoption of innovative technologies within nursing. Therefore, the aim of this systematic review was to comprehensively examine the existing literature on the role of AI in developing a future‐ready nursing workforce. By synthesizing available evidence, this review seeks to enhance understanding of how AI is being utilized within nursing contexts and to identify key directions for future research, practice, and workforce development initiatives.

## 2. Methodology

### 2.1. Study Design

This study was conducted as a systematic review to comprehensively examine the role of AI in developing a future‐ready nursing workforce. The review adhered to the Preferred Reporting Items for Systematic Reviews and Meta‐Analyses (PRISMA 2020) guidelines (Supporting File 1) to ensure methodological transparency, consistency, and rigor throughout the review process [27]. To enhance the credibility and reproducibility of the review, the study protocol was registered in PROSPERO (Registration ID: CRD420261433767) prior to the commencement of the review. The review process followed a predefined protocol that guided study identification, selection, data extraction, quality assessment, and synthesis of findings.

### 2.2. Search Strategy

A comprehensive literature search was undertaken across several electronic databases, including PubMed, Scopus, Web of Science, CINAHL, and IEEE Xplore (Table 1). These databases were selected to capture multidisciplinary evidence from nursing, health care, computer science, and health informatics literature. The search strategy combined controlled vocabulary terms and free‐text keywords related to AI and nursing workforce development. Key search terms included “artificial intelligence,” “machine learning,” “deep learning,” “nursing workforce,” “nursing education,” “clinical decision support,” “workforce readiness,” “digital health,” and “future‐ready nursing.” Boolean operators such as “AND” and “OR” were used to refine and expand the search. Reference lists of eligible articles were also manually screened to identify additional relevant studies that may not have been captured through database searches.

**TABLE 1:** Search strategy.

Database	Search string	Keywords used	Boolean operators	Filters applied	Search fields	Time frame	Study type	Notes
PubMed	(“Artificial Intelligence” [MeSH] OR “Machine Learning” [MeSH] OR “Deep Learning” [MeSH] OR “Natural Language Processing” [MeSH] OR “Decision Support Systems, Clinical” [MeSH] OR “predictive analytic∗” [tiab] OR “intelligent tutoring” [tiab] OR “adaptive learning” [tiab] OR “large language model∗” [tiab] OR “generative AI” [tiab] OR “algorithm∗” [tiab]) AND (“Nursing Staff” [MeSH] OR “Nurses” [MeSH] OR “Education, Nursing” [MeSH] OR “Professional Development” [MeSH] OR “Workforce” [MeSH] OR “Personnel Staffing and Scheduling” [MeSH] OR “nurs∗” [tiab] OR “nursing workforce” [tiab] OR “nursing education” [tiab] OR “workforce planning” [tiab] OR “staffing” [tiab]) AND (“Competency‐Based Education” [MeSH] OR “competenc∗” [tiab] OR “digital literacy” [tiab] OR “workforce readiness” [tiab] OR “future‐ready” [tiab] OR “workforce development” [tiab] OR “professional preparedness” [tiab] OR “technological readiness” [tiab])	Artificial intelligence; Machine learning; Deep learning; Natural language processing; Predictive analytics; Intelligent tutoring; Adaptive learning; Large language model; Generative AI; Algorithm; Clinical decision support; Nurses; Nursing staff; Nursing education; Nursing workforce; Workforce planning; Staffing; Professional development; Competency; Digital literacy; Workforce readiness; Future‐ready; Workforce development; Professional preparedness; Technological readiness	AND (between concept blocks) OR (within concept blocks) Wildcard: ∗ (truncation)	Publication date: January 2015–February 2026 Language: English Document type: Journal article Journal source: Yes	MeSH Terms [MeSH]‐controlled vocabulary (exploded) Title/Abstract [tiab] = free text	January 2015–February 2026	Quantitative, qualitative, and mixed methods research articles	MeSH exploded terms used to capture subject headings and subheadings. Free‐text [tiab] terms added for studies not yet indexed. Primary source for biomedical and clinical nursing/AI literature.

Scopus	TITLE‐ABS‐KEY(“artificial intelligence” OR “machine learning” OR “deep learning” OR “neural network∗” OR “natural language processing” OR “predictive analytic∗” OR “clinical decision support” OR “intelligent tutoring” OR “adaptive learning” OR “large language model∗” OR “generative AI”) AND TITLE‐ABS‐KEY (“nurs∗” OR “nursing workforce” OR “nursing education” OR “nursing practice” OR “nursing staff” OR “workforce planning” OR “staffing” OR “nursing personnel”) AND TITLE‐ABS‐KEY (“competenc∗” OR “digital literacy” OR “workforce readiness” OR “future‐ready” OR “workforce development” OR “professional preparedness” OR “technological readiness”)	Artificial intelligence; Machine learning; Deep learning; Neural network; Natural language processing; Predictive analytics; Clinical decision support; Intelligent tutoring; Adaptive learning; Large language model; Generative AI; Nursing workforce; Nursing education; Nursing practice; Nursing staff; Staffing; Workforce planning; Competency; Digital literacy; Workforce readiness; Future‐ready; Professional preparedness; Technological readiness	AND (between concept blocks) OR (within concept blocks) Wildcard: ∗ (truncation)	Publication year: 2015–2026 Language: English Document type: Article (DOCTYPE = ar) Source type: Journal	TITLE‐ABS‐KEY‐searches Title, Abstract, and Keywords simultaneously	January 2015–February 2026	Quantitative, qualitative, and mixed methods research articles	Scopus does not use MeSH; TITLE‐ABS‐KEY field used for free‐text searching. Broad multidisciplinary coverage capturing nursing, health informatics, and computer science literature.

Web of Science	TS = (“artificial intelligence” OR “machine learning” OR “deep learning” OR “neural network∗” OR “natural language processing” OR “predictive analytic∗” OR “clinical decision support” OR “intelligent tutoring” OR “adaptive learning” OR “large language model∗” OR “generative AI”) AND TS = (“nurs∗” OR “nursing workforce” OR “nursing education” OR “nursing practice” OR “nursing staff” OR “staffing” OR “workforce planning”) AND TS = (“competenc∗” OR “digital literacy” OR “workforce readiness” OR “future‐ready” OR “workforce development” OR “professional preparedness” OR “technological readiness”)	Artificial intelligence; Machine learning; Deep learning; Neural network; Natural language processing; Predictive analytics; Clinical decision support; Intelligent tutoring; Adaptive learning; Large language model; Generative AI; Nursing workforce; Nursing education; Nursing practice; Nursing staff; Staffing; Workforce planning; Competency; Digital literacy; Workforce readiness; Future‐ready; Professional preparedness; Technological readiness	AND (between concept blocks) OR (within concept blocks) Wildcard: ∗ (truncation)	Publication year: 2015–2026 Language: English Document type: Article Web of Science Core Collection	TS = Topic field (searches Title, Abstract, Author Keywords, and Keywords Plus simultaneously)	January 2015–February 2026	Quantitative, qualitative, and mixed methods research articles	TS (Topic) field provides broad coverage including Keywords Plus terms. Strong coverage of high‐impact journals. Citation tracking used for additional records.

CINAHL	((MH “Artificial Intelligence”) OR (MH “Machine Learning”) OR (TI, AB (“artificial intelligence” OR “machine learning” OR “algorithm∗” OR “deep learning” OR “natural language processing” OR “predictive analytic∗” OR “clinical decision support” OR “intelligent tutoring” OR “adaptive learning” OR “large language model∗”))) AND ((MH “Nurses”) OR (MH “Nursing Staff”) OR (MH “Education, Nursing”) OR (TI,AB(“nurs∗” OR “nursing workforce” OR “nursing education” OR “nursing practice” OR “workforce planning” OR “staffing”))) AND (TI,AB(“competenc∗” OR “digital literacy” OR “workforce readiness” OR “future‐ready” OR “workforce development” OR “professional preparedness” OR “technological readiness”))	Artificial intelligence; Machine learning; Algorithm; Deep learning; Natural language processing; Predictive analytics; Clinical decision support; Intelligent tutoring; Adaptive learning; Large language model; Nurses; Nursing staff; Nursing education; Nursing workforce; Nursing practice; Staffing; Workforce planning; Competency; Digital literacy; Workforce readiness; Future‐ready; Professional preparedness; Technological readiness	AND (between concept blocks) OR (within concept blocks) Wildcard: ∗ (truncation)	Publication date: 2015–present Language: English Document type: Journal article Journal source limiter: Yes Exclude MEDLINE records	MH = CINAHL Subject Headings (controlled vocabulary) TI, AB = Title and Abstract free‐text fields	January 2015–February 2026	Quantitative, qualitative, and mixed methods research articles	CINAHL Subject Headings (MH) specific to nursing and allied health. Gold‐standard database for nursing literature. Journal‐source limiter and MEDLINE exclusion applied to minimize duplication.

IEEE Xplore	(“All Metadata”: “artificial intelligence” OR “All Metadata”: “machine learning” OR “All Metadata”: “deep learning” OR “All Metadata”: “natural language processing” OR “All Metadata”: “clinical decision support” OR “AllMetadata”: “predictive analytics” OR “All Metadata”: “intelligent tutoring” OR “All Metadata”: “adaptive learning” OR “All Metadata”: “large language model∗”) AND (“All Metadata”: “nursing” OR “All Metadata”: “nurse” OR “All Metadata”: “nursing education” OR “All Metadata”: “healthcare workforce” OR “All Metadata”: “clinical training” OR “All Metadata”: “workforce development”) AND (“All Metadata”: “competency” OR “All Metadata”: “digital literacy” OR “AllMetadata”: “workforce readiness” OR “All Metadata”: “health informatics”)	Artificial intelligence; Machine learning; Deep learning; Natural language processing; Clinical decision support; Predictive analytics; Intelligent tutoring; Adaptive learning; Large language model; Nursing; Nurse; Nursing education; Healthcare workforce; Clinical training; Workforce development; Competency; Digital literacy; Workforce readiness; Health informatics	AND (between concept blocks) OR (within concept blocks) Wildcard: ∗ (truncation)	Publication year: 2015–2026 Language: English Document type: Journals and Conference Papers	“All Metadata”‐searches all available metadata fields including title, abstract, and index terms	January 2015–February 2026	Quantitative, qualitative, and mixed methods research articles	Captures AI and technology‐focused research relevant to healthcare and nursing informatics. Complements clinical databases by covering computer science and engineering perspectives.

Manual/reference list screening	Hand‐search of reference lists of all included studies and forward citation tracking of key included articles via Scopus and Web of Science	Not applicable‐manual citation tracking	Not applicable	Journal articles only Published: 2015–2026 Language: English	Reference lists of all included full‐text articles; forward citation tracking	January 2015–February 2026	Quantitative, qualitative, and mixed methods research articles	Reference lists of all studies meeting full inclusion criteria were manually screened by two reviewers. Forward citation tracking conducted for key included articles. Aims to identify eligible publications not captured by database searches.

### 2.3. Eligibility Criteria

Studies were selected according to predefined inclusion and exclusion criteria. Articles were eligible if they were primary empirical studies examining the application, impact, implementation, evaluation, perceptions, readiness, or potential of an identifiable AI technology or AI‐specific construct in nursing education, nursing practice, workforce development, competency enhancement, workforce planning, or professional preparedness. Quantitative, qualitative, and mixed methods studies were eligible. Journal articles published in English between January 2015 and February 2026 were included. Studies were excluded if they focused on other healthcare professions without specific nursing relevance, did not contain an identifiable AI or algorithmic component or AI‐specific construct, or addressed only general leadership, resilience, turnover, missed care, staffing, or organizational variables without an AI linkage. Editorials, commentaries, conference abstracts, dissertations, reviews, theoretical or conceptual papers, and reports lacking sufficient methodological detail were excluded from the primary synthesis.

### 2.4. Study Selection Process

All retrieved records were exported into reference management software and duplicate citations were removed before screening. Titles and abstracts were independently screened by two reviewers, followed by independent full‐text assessment of potentially eligible reports. Disagreements were resolved through discussion and, when required, consultation with a third reviewer. Full‐text eligibility assessment applied the prespecified AI‐specific criteria consistently. Thirteen studies were excluded because they did not involve an AI technology or AI‐specific construct, and one review/conceptual paper was excluded because nonprimary studies were ineligible. The final primary synthesis comprised 38 studies. The selection process is summarized in the PRISMA flow diagram (Figure 1).

**FIGURE 1:**
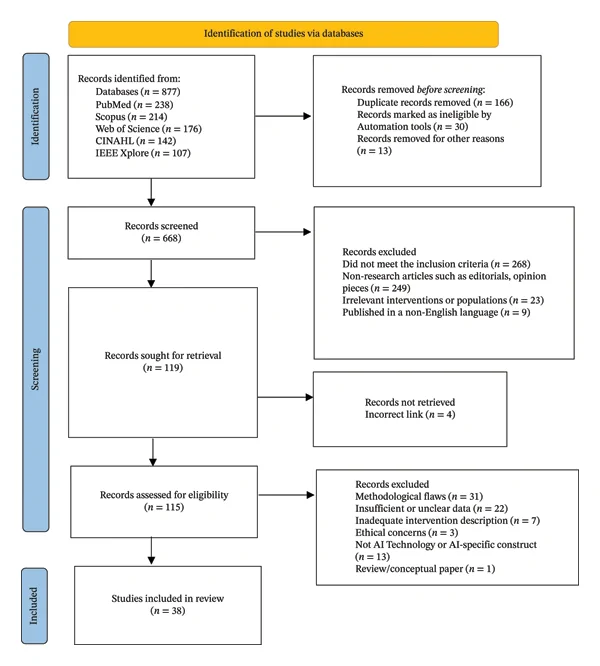
PRISMA flow diagram.

### 2.5. Data Extraction

A standardized data extraction form was developed to ensure consistency across studies. Information extracted from each included articles comprised of author details, year of publication, country of study, study design, sample characteristics, AI technologies employed, nursing‐related outcomes, key findings, and reported benefits or challenges associated with AI implementation. Additional information regarding educational interventions, workforce competencies, clinical applications, and organizational impacts was also collected where available. Data extraction was performed independently by two reviewers, and extracted information was cross‐checked to ensure accuracy and completeness.

### 2.6. Quality Assessment

Methodological quality was assessed using the Mixed Methods Appraisal Tool (MMAT) 2018 because the eligible evidence included qualitative, quantitative, and mixed methods designs (Table 2) [66]. Eligibility and methodological quality were treated as separate steps: MMAT appraisal was applied only after a study satisfied the review’s AI‐specific inclusion criteria. Thus, a methodologically sound study without an AI component was not considered eligible for this review. Each of the 38 studies was assessed using the relevant MMAT criteria, including research‐question clarity, appropriateness of design and sampling, adequacy of data collection and outcome measurement, and analytical rigor. Two reviewers appraised studies independently and resolved discrepancies through discussion. Appraisal findings were used to contextualize evidence strength rather than as a basis for posteligibility exclusion.

**TABLE 2:** MMAT quality assessment.

References	Author(s)	Research questions clear?	Data address research questions?	Study design appropriate?	Data collection methods adequate?	Findings adequately derived from data?	Score and overall quality
[28]	Rony et al., 2025	Yes	Yes	Yes	Partial	Yes	High
[29]	El‐Bassal et al., 2025	Yes	Yes	Yes	Partial	Yes	High
[30]	Ghimire & Qiu, 2025	Yes	Yes	Yes	Yes	Partial	High
[31]	Alruwaili et al., 2024	Yes	Partial	Partial	Partial	Partial	Low
[32]	Chu et al., 2026	Yes	Yes	Yes	Yes	Yes	High
[33]	Benfatah et al., 2026	Yes	Yes	Yes	Partial	Yes	High
[34]	Harder et al., 2025	Yes	Partial	Yes	Partial	Yes	Moderate
[35]	Göktepe & Sarıköse, 2025	Yes	Yes	Yes	Yes	Yes	High
[36]	Atalla et al., 2025	Yes	Partial	Yes	Partial	Partial	Moderate
[37]	Shorey et al., 2019	Yes	Yes	Yes	Yes	Yes	High
[38]	Kotp et al., 2025	Yes	Yes	Yes	Yes	Yes	High
[39]	Alnomasy et al., 2025	Yes	Yes	Yes	Partial	Yes	High
[40]	Alruwaili et al., 2025	Yes	Yes	Yes	Yes	Yes	High
[41]	Lukkien et al., 2024	Yes	Yes	Yes	Yes	Yes	High
[42]	Levin et al., 2025	Yes	Partial	Yes	Partial	Partial	Moderate
[43]	Latif et al., 2020	Yes	Yes	Partial	Partial	Partial	Moderate
[44]	Karnehed et al., 2025	Yes	Yes	Yes	Yes	Yes	High
[45]	Clej et al., 2026	Yes	Yes	Yes	Yes	Yes	High
[46]	Hah & Goldin, 2021	Yes	Partial	Yes	Partial	Partial	Moderate
[47]	Salama et al., 2025	Yes	Partial	Partial	Partial	Partial	Low
[48]	Liu et al., 2026	Yes	Yes	Partial	Yes	Yes	High
[49]	Saban & Dubovi, 2025	Yes	Partial	Yes	Partial	Yes	Moderate
[50]	Bodur et al., 2025	Yes	Yes	Yes	Partial	Yes	High
[51]	Ortiz‐Barrios et al., 2025	Yes	Yes	Yes	Partial	Yes	High
[52]	Shoaib et al., 2026	Yes	Partial	Yes	Partial	Partial	Moderate
[53]	Liao et al., 2015	Yes	Yes	Yes	Yes	Yes	High
[54]	Lukić et al., 2023	Yes	Yes	Yes	Yes	Yes	High
[55]	Wang et al., 2024	Yes	Yes	Yes	Yes	Yes	High
[56]	Yıldırım et al., 2026	Yes	Yes	Yes	Yes	Yes	High
[57]	Cho & Seo, 2024	Yes	Yes	Yes	Yes	Yes	High
[58]	Yıldırım & Karaman, 2025	Yes	Yes	Yes	Yes	Yes	High
[59]	Liaw et al., 2023	Yes	Yes	Yes	Yes	Yes	High
[60]	Ergin et al., 2022	Yes	Partial	Yes	Partial	Partial	Moderate
[61]	Almagharbeh et al., 2025	Yes	Yes	Yes	Yes	Yes	High
[62]	Hassan & El‐Ashry, 2024	Yes	Yes	Yes	Yes	Yes	High
[63]	Sommer et al., 2024	Yes	Yes	Yes	Yes	Yes	High
[64]	Han et al., 2022	Yes	Yes	Yes	Yes	Yes	High
[65]	Kwak et al., 2022	Yes	Yes	Yes	Yes	Yes	High

### 2.7. Data Synthesis

A narrative synthesis was used because of substantial heterogeneity in study designs, AI applications, outcomes, and evaluation methods. Only findings directly linked to an AI technology or to AI‐specific perceptions, readiness, literacy, or implementation were coded into the synthesis. General findings on leadership, resilience, missed care, turnover, staffing, or work environment from studies without an AI component were not used to infer AI effects. The synthesis distinguished intervention or performance outcomes from self‐reported perceptions and readiness measures, and it considered whether findings came from real‐world implementation, simulation, prototype development, or cross‐sectional assessment. Themes included education and training, clinical decision support, workforce planning, competency and digital readiness, organizational implications, and implementation barriers (Figure 2).

**FIGURE 2:**
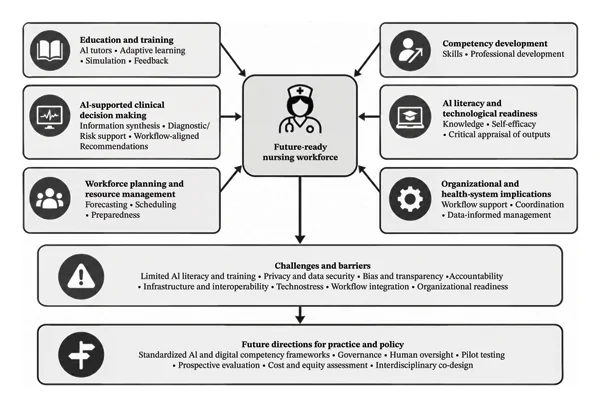
Potential AI contributions to a future‐ready nursing workforce.

## 3. Results

### 3.1. Summary of Included Studies

A total of 38 primary empirical studies met the eligibility criteria [28–65] and were included in the systematic review (Supporting File 2). The studies were published between 2015 and 2026 and represented a methodologically heterogeneous evidence base. Cross‐sectional studies were most common (*n* = 15), followed by qualitative studies (*n* = 11), development or prototype studies (*n* = 6), mixed methods studies (*n* = 3), randomized or quasi‐experimental studies (*n* = 2), and one methodological study. Geographically, Turkey (*n* = 5), Egypt (*n* = 4), Saudi Arabia (*n* = 4), and South Korea (*n* = 3) were the most frequently represented settings; China, Canada, Singapore, and multicountry studies contributed two studies each, with the remaining 14 studies distributed across other countries (Figure 3).

**FIGURE 3:**
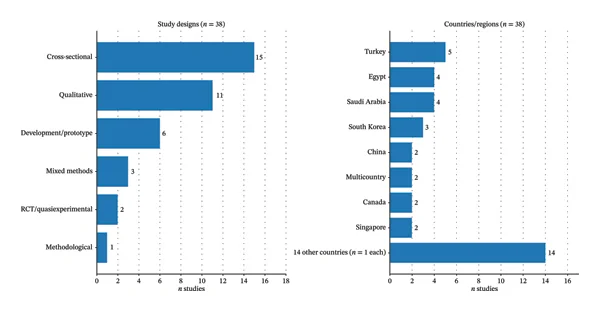
Distribution of study designs and geographic representation (*n* = 38).

The included studies examined machine learning and predictive analytics, generative AI and large language models, AI‐supported clinical decision systems, virtual patients and simulation, conversational agents, AI‐enabled learning systems, and AI‐related readiness and literacy (Table 3). The evidence suggested potential benefits for personalized learning, clinical reasoning support, staffing forecasting, professional development, and workflow assistance, but the strength of evidence varied substantially. Many studies assessed attitudes, perceived readiness, or prototype performance rather than sustained clinical or organizational outcomes. Table 3 therefore provides a technology‐level thematic synthesis with representative studies rather than an exhaustive list of all included records; the complete 38‐study inventory is provided in Supporting File 2. Recurrent concerns included AI literacy gaps, privacy and ethical issues, bias and explainability, infrastructure limitations, technostress, and organizational preparedness.

**TABLE 3:** Representative AI technologies, nursing applications, and illustrative included studies.

AI technology type	Nursing application domain	Representative studies	Key reported outcomes	Notable challenges
Intelligent tutoring systems/adaptive learning platforms	Nursing education; personalized learning; course‐specific tutoring	Ghimire & Qiu [30]; Chu et al. [32]	Personalized learning, feedback, engagement, conceptual understanding, and support for self‐directed learning; evidence includes student perceptions and a mixed methods virtual‐tutor evaluation	Evidence often based on short‐term, self‐reported, or single‐program evaluations; risk of over‐reliance
AI‐enhanced virtual reality/simulation	Clinical skills training; communication; diagnostic reasoning; interprofessional education	Harder et al. [34]; Shorey et al. [37]; Hah & Goldin [46]; Liaw et al. [59]	Improved or perceived gains in communication, confidence, knowledge practice, and low‐risk iterative learning; evidence spans qualitative, development, and mixed methods designs	Limited number of comparative studies; short follow‐up; reduced emotional or physical realism in some systems
Generative AI/large language models	Clinical decision support; triage; neonatal care; vignette‐based reasoning	Alruwaili et al. [40]; Levin et al. [42]; Saban & Dubovi [49]	Rapid information synthesis and decision support, but variable agreement with nursing judgment, over‐triage, and a continuing need for contextual interpretation and human oversight	Variable agreement with professional judgment; over‐triage or inappropriate recommendations; privacy and uncritical‐use risks
Clinical decision support systems/diagnostic AI	Patient monitoring; risk assessment; diagnostic support; nursing diagnosis; remote decision support	Lukkien et al. [41]; Clej et al. [45]; Hah & Goldin [46]; Liao et al. [53]; Yildirim et al. [56]; Hassan & El‐Ashry [62]	Support for decision making, diagnosis, early identification of care needs, and monitoring; adoption depends on eHealth literacy, workflow fit, trust, and accountability	Alert burden, explainability, accountability, workflow disruption, technostress, and dependence on local implementation
Predictive analytics/machine learning	Workforce planning; staffing and scheduling; demand forecasting; resource allocation	Göktepe & Sarıköse [35]; Kotp et al. [38]; Ortiz‐Barrios et al. [51]; Shoaib et al. [52]	Potential for staffing prediction, scheduling optimization, and managerial planning; strongest direct operational evidence comes from modeling and prototype studies	Much evidence is model‐, prototype‐, or perception‐based; limited prospective and multi‐site validation
AI chatbots/conversational agents	Prebriefing; communication training; educational feedback; fetal‐monitoring education	Benfatah et al. [33]; Shorey et al. [37]; Han et al. [64]	Personalized prebriefing and flexible practice; communication training and feedback; improved preparation or engagement, although effects on knowledge and clinical reasoning were not uniformly significant	Open‐ended dialog limitations, novelty effects, variable realism, and risk of dependency
AI‐enabled monitoring/image recognition/remote care	Home monitoring; wound assessment; ICU monitoring; early warning and medication support	Latif et al. [43]; Karnehed et al. [44]; Almagharbeh et al. [61]; Hassan & El‐Ashry [62]	Continuous monitoring, wound‐analysis support, early warning, and workflow assistance; evidence remains prototype, pilot, or perception based in several settings	Data quality and security; interoperability; workflow fit; patient access and digital‐divide concerns

### 3.2. Artificial Intelligence in Nursing Education and Training

AI was most frequently examined in educational and training contexts, but the outcomes and level of evaluation varied. Studies of AI tutors, generative AI tools, simulation, and adaptive learning described improved access to feedback, personalization, engagement, and perceived preparedness [30, 32, 33, 37, 64]. Development and mixed methods further showed that virtual tutors and AI‐supported learning environments could provide repeated practice, interactive learning, and formative feedback, though several evaluations relied on acceptability, perceptions, or short‐term outcomes rather than sustained competence [32, 37, 46, 59]. These findings support the educational potential of AI but do not establish equivalent effectiveness across curricula, institutions, or learner groups.

Simulation‐focused evidence provided more direct evaluation. Randomized and mixed methods studies reported gains in preparation, confidence, communication knowledge, or engagement [33, 59], while qualitative comparison suggested lower performance anxiety and benefits of repeated practice with AI‐enhanced virtual reality [34]. In contrast, one quasi‐experimental chatbot study increased educational interest but did not significantly improve knowledge, clinical reasoning competency, or confidence [64], and an AI‐enabled diagnostic simulation study did not significantly enhance diagnostic capability [46]. Accordingly, AI‐supported education is better interpreted as a promising adjunct to educator‐led teaching and simulation than as a replacement for supervised clinical learning.

### 3.3. AI‐Supported Clinical Decision Making

Clinical decision‐support studies examined generative AI, algorithmic decision aids, diagnostic support, and AI‐enabled information processing [40–43, 46, 49, 53]. Nurses and clinicians often valued rapid information synthesis and decision support, particularly when systems were transparent and aligned with workflow. At the same time, comparative and qualitative studies identified important limitations, including inappropriate prioritization, variable agreement with professional judgment, uncertainty about accountability, and the need for contextual interpretation [41, 42, 49]. These findings indicate that AI can augment information processing, but performance in a constrained vignette, prototype, or single‐site setting should not be equated with safe autonomous decision making in routine care.

Human oversight remained a consistent requirement. Studies of AI‐assisted decision making and willingness to use AI systems showed that trust, eHealth literacy, explainability, and perceived usefulness influenced adoption [41, 45, 46, 50, 62]. Technostress and concerns about over‐reliance may offset efficiency gains when systems are poorly integrated into nursing workflows [45]. Therefore, the evidence supports a human‐in‐the‐loop model in which AI recommendations are treated as decision support and remain subject to nursing assessment, ethical reasoning, and patient‐specific judgment [40–42, 49, 56, 62].

### 3.4. Workforce Planning and Resource Management

Evidence for AI in workforce planning was limited and less mature than evidence in education and clinical decision support. Eligible studies used predictive analytics and AI‐enabled human‐resource systems to forecast workload, staffing demand, scheduling needs, or workforce preparedness [51, 52]. These studies indicate that data‐driven forecasting may improve planning precision and help managers explore staffing scenarios. However, much of this evidence arose from modeling, prototype systems, or cross‐sectional perceptions rather than prospective organization‐wide implementation.

Importantly, the evidence base does not support attributing nurse retention, resilience, burnout reduction, or lower missed‐care rates to AI when those outcomes were not evaluated in AI‐specific studies. No eligible longitudinal study demonstrated that AI itself improved retention or workforce resilience. Claims regarding organizational workforce outcomes have therefore been restricted to directly evaluated AI‐related forecasting, scheduling, preparedness, and managerial decision‐support functions [35, 38, 39, 51, 52, 60].

### 3.5. Competency Development, AI Literacy, and Technological Readiness

Competency development and digital readiness were closely overlapping themes and were therefore consolidated. Cross‐sectional and qualitative studies consistently showed that nurses and nursing students perceived AI knowledge, literacy, self‐efficacy, and confidence as important prerequisites for effective use [29, 31, 36, 38, 39, 47, 48, 54–57, 63, 65]. Exposure to AI tools was often associated with greater familiarity and willingness to engage with technology, but readiness measures primarily reflect attitudes or self‐perceived capability rather than verified clinical competence [31, 47, 54–57, 63, 65].

The evidence also showed substantial variation in confidence and acceptance. Anxiety, limited understanding of AI outputs, ethical concerns, and uncertainty about changing professional roles could reduce intention to use AI even when perceived usefulness was high [31, 54, 57, 63, 65]. This pattern suggests that competency initiatives should combine technical skills with data literacy, critical appraisal of algorithmic outputs, ethics, communication, and escalation procedures rather than focusing on tool operation alone [28, 30, 35, 36, 41, 42, 48, 50, 56, 61–63, 65].

### 3.6. Organizational and Healthcare System Implications

Eligible studies described possible organizational advantages such as workflow support, improved access to information, better coordination, and more data‐informed management [35, 39, 50–53, 60–62]. Nurse managers also identified potential for AI to assist administrative and operational decisions [35, 39, 60]. Nevertheless, much of this evidence was perceptual, qualitative, or prototype‐based, so reported benefits should not be interpreted as established reductions in workload, cost, or staffing pressure across healthcare systems.

Organizational value appeared contingent on implementation conditions. Interoperability, usable interfaces, local data quality, staff involvement, leadership support, and clear accountability were repeatedly identified as prerequisites for effective adoption [35, 40, 41, 44–46, 50–52, 61, 62]. These findings shift the emphasis from whether AI is technically capable to whether an organization can integrate, govern, monitor, and sustain the technology without introducing new workload, safety, or equity risks.

### 3.7. Challenges and Barriers to Adoption of AI

Across the evidence base, limited AI literacy and training were among the most consistent barriers [28–31, 36, 38, 39, 47, 48, 50, 54–57, 61–63, 65]. Nurses reported uncertainty about how AI systems generate recommendations, when outputs should be challenged, and how responsibility should be shared between clinicians and technology. Acceptance was also shaped by perceived usefulness, anxiety, trust, technostress, and prior digital experience [31, 41, 45, 54, 56, 57, 62, 63, 65].

Ethical and organizational concerns included privacy, data security, bias, transparency, accountability, professional autonomy, infrastructure readiness, and workflow disruption [28, 30, 31, 35, 40–42, 44–46, 50, 55, 56, 58, 60–63, 65]. These barriers are interconnected: weak infrastructure can undermine performance, limited explainability can reduce trust, and inadequate training can increase both misuse and resistance. Sustainable implementation therefore requires governance, workforce preparation, technical validation, and organizational change management to proceed together rather than as separate activities.

### 3.8. Future Directions for a Future‐Ready Nursing Workforce

Future research should prioritize multicenter and prospective evaluations that compare AI‐supported care or education with appropriate alternatives and report clinically or educationally meaningful outcomes, not only attitudes or usability [33, 34, 46, 51, 52, 64]. Standardized measures of AI literacy, digital competence, trust, workflow impact, safety, equity, and implementation outcomes would improve comparability [29, 38, 39, 45, 48, 58, 63, 65]. Studies should also distinguish between model performance, user acceptance, and real‐world effectiveness because these represent different levels of evidence [32, 46, 51, 52].

For workforce development, a central priority is the establishment of standardized AI and digital competency frameworks for students, frontline nurses, and nursing leaders [28–30, 35–39, 47, 48, 54–57, 60–63, 65]. Such frameworks should include technical literacy, critical evaluation of outputs, data governance, ethical reasoning, bias awareness, communication with patients, and procedures for human oversight [28, 30, 32, 35, 36, 40–42, 45, 48, 50, 56, 58, 61–63, 65]. Interdisciplinary codesign with nurses, educators, informaticians, patients, regulators, and technology developers is needed so that future systems fit nursing workflows and professional responsibilities rather than requiring nurses to adapt to poorly aligned technologies [30, 35, 40–42, 44, 46, 50, 61–63].

## 4. Discussion

The 38 included studies show that AI is being explored across education, clinical decision support, readiness, and workforce planning, but the maturity of evidence is uneven. Cross‐sectional and qualitative studies dominate, whereas comparative, longitudinal, and implementation studies are scarce. This distinction is important because positive attitudes, prototype accuracy, or short‐term simulation gains do not demonstrate durable improvements in clinical outcomes, workforce sustainability, or organizational performance. Accordingly, the synthesis treats AI as an enabling technology whose effects depend on context, design quality, workflow fit, and human oversight rather than as an independent solution to nursing workforce challenges.

Educational findings illustrate this tension. Included studies suggest that AI tutors, virtual patients, and AI‐enhanced simulation can support feedback, engagement, and practice [30, 32–34, 37, 59, 64]. However, a recent systematic review and meta‐analysis published after the review’s search window found improvements in nursing students’ knowledge and satisfaction but high heterogeneity and less consistent effects on clinical reasoning or performance [67]. Similarly, quasi‐experimental evidence comparing AI‐assisted assessment with standardized‐patient approaches reported high agreement between AI and human ratings of therapeutic communication, but this does not remove the need for validation across institutions, languages, and communication contexts [68]. These external studies are discussed as contextual evidence and were not counted among the 38 studies identified through prespecified systematic search.

Clinical and workforce applications show a similar gap between technical promises and implementation evidence. Generative and algorithmic decision‐support systems can synthesize information rapidly, yet comparative work has also shown variable agreement with nursing judgment and the possibility of over‐triage or inappropriate recommendations [42, 49]. Technostress, limited eHealth literacy, and concerns about accountability may create new burdens when tools are poorly integrated [45]. In workforce planning, predictive systems can forecast staffing or scheduling requirements [51, 52], but no eligible longitudinal evidence demonstrated that AI itself improves retention, resilience, burnout, or missed care. The evidence therefore supports a narrower interpretation: AI may support forecasting and managerial decisions, but broader workforce benefits remain hypotheses requiring direct prospective evaluation.

Implementation of readiness may be the most immediate determinant of whether these technologies help or hinder nursing practice. Included studies and recent external evidence show interest in AI alongside persistent gaps in knowledge, confidence, governance, and organizational preparedness [29, 31, 36, 38, 39, 55–57, 63, 69]. Success is therefore likely to depend on more than model accuracy. Interoperability, local data quality, cybersecurity, explainability, training time, leadership support, procurement decisions, and postdeployment monitoring can determine whether a technically promising system becomes usable and safe. Failures and nonsignificant outcomes may also be less visible in the published literature. Future evaluation should therefore report implementation problems and unintended consequences as systematically as benefits, and should include economic, equity, and workflow outcomes alongside conventional performance metrics.

## 5. Implications for Practice and Policy

For nursing practice and education, AI should be introduced as a supervised support resource rather than an autonomous substitute for professional judgment. Nursing programs and employers should adopt standardized AI and digital competency frameworks covering data literacy, interpretation of algorithmic outputs, bias and uncertainty, privacy, ethics, patient communication, and escalation when AI advice conflicts with clinical assessment. Implementation should begin with defined use cases and staged pilots that include frontline nurses in codesign, training, and evaluation. Pilot outcomes should include not only accuracy or satisfaction but also workload, safety, equity, usability, and effects on patient‐centered care.

Policy and management decisions should also address economic viability and resource allocation. Before scale‐up, organizations should assess total cost of ownership, including procurement or licensing, devices and network capacity, interoperability work, cybersecurity, staff training and backfill, technical support, model monitoring, maintenance, and replacement or upgrade costs. Budget‐impact and cost‐effectiveness evidence is especially important for public and resource‐limited health systems, where investment in AI may compete with basic staffing, education, and infrastructure needs. Policymakers should support interoperable standards, transparent procurement, data governance, algorithmic accountability, and equity‐focused evaluation, with continued funding contingent on demonstrable clinical or operational value rather than technological novelty.

## 6. Strengths and Limitations

This review draws on multiple multidisciplinary databases, PRISMA 2020 reporting, independent screening, MMAT appraisal, and explicit AI‐specific eligibility criteria. A major strength is the clear separation of AI‐related evidence from general nursing workforce literature. Restricting the synthesis to eligible primary AI studies reduced conceptual confounding and enabled a more transparent distinction between direct intervention evidence, prototype or simulation evidence, and perceptions or readiness research.

Several limitations remain. The 38 included studies were heterogeneous in design, setting, AI technology, and outcomes, preventing quantitative pooling and limiting direct comparison. The evidence was dominated by cross‐sectional, qualitative, and development studies, with little longitudinal or prospective implementation research. Restriction to English‐language publications may have excluded relevant evidence, and many studies were conducted in single institutions or specific national contexts. Publication bias is also plausible because studies reporting positive AI performance, acceptance, or implementation may be more likely to appear in the literature than studies reporting null findings, failures, or discontinued deployments; the heterogeneity of designs and outcomes precluded formal funnel‐plot assessment. Finally, rapid AI development means that evidence may become outdated quickly, particularly for generative AI systems.

## 7. Conclusion

This systematic review indicates that AI has promising but uneven potential to contribute to a future‐ready nursing workforce through education, clinical decision support, workforce forecasting, and development of AI‐related competencies. The evidence is not yet sufficiently mature to justify broad claims that AI improves retention, resilience, workload, or organizational performance, because much of the literature remains cross‐sectional, qualitative, prototype‐based, or short‐term. AI should therefore complement, not replace, nursing expertise and should be introduced through staged, human‐supervised implementation. An immediate priority is the establishment of standardized AI and digital competency frameworks for nursing leadership, educators, students, and frontline nurses, coupled with governance, predeployment validation, cost and equity assessment, and monitoring postdeployment. Future research should emphasize prospective, multicenter, and long‐term evaluation of effectiveness, safety, workflow impact, and workforce outcomes.

## Funding

There was no external fund taken for this current research.

## Conflicts of Interest

The authors declare no conflicts of interest.

## Supporting Information

Additional supporting information can be found online in the Supporting Information section.

## Supporting information


**Supporting Information 1** Supporting File 1: PRISMA 2020 Checklist. The completed PRISMA 2020 checklist presents the reporting items, corresponding locations in the main text, and reporting status to ensure transparency and completeness of the systematic review.

**Supporting Information 2** Supporting File 2: Overview of Included Studies. A comprehensive summary of the 38 studies included in the systematic review, detailing study characteristics, artificial intelligence technologies, nursing‐related outcomes, key findings, reported benefits and challenges, educational interventions, workforce competencies, clinical applications, and organizational impacts.

## Data Availability

All study‐level data analyzed in this systematic review are available in Supporting File 2.
