# Supplementary material for: The Role of Artificial Intelligence in Developing a Future‐Ready Nursing Workforce: A Systematic Review
Source: J Nurs Manag. 2026 Nov 1;2026:8272597. doi: 10.1155/jonm/8272597 (PMC13725111; doi:10.1155/jonm/8272597)
Supplement: Supplementary file 1 [file JONM-2026-8272597-s001.docx]

**Supplementary File 1: PRISMA 2020 Checklist**

| **Section and topic** | **Item** | **PRISMA 2020 checklist item** | **Location in manuscript** | **Status** |
| --- | --- | --- | --- | --- |
| TITLE | | | | |
| Title | 1 | Identify the report as a systematic review. | Page 1, title | Reported |
| ABSTRACT | | | | |
| Abstract | 2 | See the PRISMA 2020 for Abstracts checklist. | Page 2, Abstract | Reported |
| INTRODUCTION | | | | |
| Rationale | 3 | Describe the rationale for the review in the context of existing knowledge. | Pages 3-4, Introduction | Reported |
| Objectives | 4 | Provide an explicit statement of the objective(s) or question(s) the review addresses. | Page 4, final paragraph of Introduction | Reported |
| METHODS | | | | |
| Eligibility criteria | 5 | Specify the inclusion and exclusion criteria for the review and how studies were grouped for the syntheses. | Pages 5 and 7, Sections 2.3 and 2.7 | Reported |
| Information sources | 6 | Specify all databases, registers, websites, organisations, reference lists and other sources searched or consulted to identify studies. Specify the date when each source was last searched or consulted. | Page 5, Section 2.2; Table 1. | Reported |
| Search strategy | 7 | Present the full search strategies for all databases, registers and websites, including any filters and limits used. | Page 5, Section 2.2; Table 1 Search strategy | Reported |
| Selection process | 8 | Specify the methods used to decide whether a study met the inclusion criteria of the review, including how many reviewers screened each record and each report retrieved, whether they worked independently, and if applicable, details of automation tools used in the process. | Page 6, Section 2.4; Figure 1. | Reported |
| Data collection process | 9 | Specify the methods used to collect data from reports, including how many reviewers collected data from each report, whether they worked independently, any processes for obtaining or confirming data from study investigators, and if applicable, details of automation tools used in the process. | Page 6, Section 2.5 | Reported |
| Data items | 10a | List and define all outcomes for which data were sought. Specify whether all results that were compatible with each outcome domain in each study were sought, and if not, the methods used to decide which results to collect. | Page 6, Section 2.5; Supplementary file 2 | Reported |
| Data items | 10b | List and define all other variables for which data were sought. Describe any assumptions made about any missing or unclear information. | Page 6, Section 2.5; Supplementary file 2. Extracted variables are specified; assumptions for missing or unclear information are not described. | Partially reported |
| Study risk of bias assessment | 11 | Specify the methods used to assess risk of bias in the included studies, including details of the tool(s) used, how many reviewers assessed each study and whether they worked independently, and if applicable, details of automation tools used in the process. | Page 7, Section 2.6; Table 2 MMAT quality assessment | Reported |
| Effect measures | 12 | Specify for each outcome the effect measure(s) used in the synthesis or presentation of results. | Not applicable; the findings were synthesized narratively and no common pooled effect measure was used. | Not applicable |
| Synthesis methods | 13a | Describe the processes used to decide which studies were eligible for each synthesis. | Pages 5 and 7, Sections 2.3 and 2.7 | Reported |
| Synthesis methods | 13b | Describe any methods required to prepare the data for presentation or synthesis, such as handling of missing summary statistics or data conversions. | Not applicable; no quantitative data conversion or preparation for meta-analysis was required. | Not applicable |
| Synthesis methods | 13c | Describe any methods used to tabulate or visually display results of individual studies and syntheses. | Pages 8-11, Sections 3.1-3.8; Figures 2-3; Table 3; Supplementary file 2 | Reported |
| Synthesis methods | 13d | Describe any methods used to synthesize results and provide a rationale for the choice(s). If meta-analysis was performed, describe the model(s), heterogeneity methods, and software used. | Page 7, Section 2.7 | Reported |
| Synthesis methods | 13e | Describe any methods used to explore possible causes of heterogeneity among study results. | No formal investigation of causes of heterogeneity was conducted; heterogeneity informed the narrative synthesis. | Not applicable |
| Synthesis methods | 13f | Describe any sensitivity analyses conducted to assess robustness of the synthesised results. | Not applicable; no sensitivity analysis was conducted. | Not applicable |
| Reporting bias assessment | 14 | Describe any methods used to assess risk of bias due to missing results in a synthesis (arising from reporting biases). | No formal assessment of reporting bias due to missing results was performed. | Not assessed |
| Certainty assessment | 15 | Describe any methods used to assess certainty (or confidence) in the body of evidence for an outcome. | No formal certainty-of-evidence framework was applied. | Not assessed |
| RESULTS | | | | |
| Study selection | 16a | Describe the results of the search and selection process, from the number of records identified in the search to the number of studies included in the review, ideally using a flow diagram. | Page 6, Section 2.4 and Figure 1; page 8, Section 3.1 | Reported |
| Study selection | 16b | Cite studies that might appear to meet the inclusion criteria, but which were excluded, and explain why they were excluded. | Page 6, Section 2.4; Supplementary file 2, eligibility exclusions | Reported |
| Study characteristics | 17 | Cite each included study and present its characteristics. | Page 8, Section 3.1; Supplementary file 2 | Reported |
| Risk of bias in studies | 18 | Present assessments of risk of bias for each included study. | Page 7, Section 2.6; Table 2 MMAT quality assessment | Reported |
| Results of individual studies | 19 | For all outcomes, present, for each study: (a) summary statistics for each group where appropriate / (b) an effect estimate and its precision, ideally using structured tables or plots. | Pages 8-11, Sections 3.1-3.8; Table 3; Supplementary file 2. Study-level findings are presented narratively. | Reported |
| Results of syntheses | 20a | For each synthesis, briefly summarise the characteristics and risk of bias among contributing studies. | Pages 8-11, Sections 3.1-3.8; Table 2; Supplementary file 2. Methodological quality is reported, but risk of bias is not summarized separately for each thematic synthesis. | Reported |
| Results of syntheses | 20b | Present results of all statistical syntheses conducted. If meta-analysis was done, present summary estimates, precision, heterogeneity, and direction of effect where applicable. | Not applicable; no meta-analysis or other statistical synthesis was performed. | Not applicable |
| Results of syntheses | 20c | Present results of all investigations of possible causes of heterogeneity among study results. | Not applicable; no formal investigation of causes of heterogeneity was conducted. | Not applicable |
| Results of syntheses | 20d | Present results of all sensitivity analyses conducted to assess the robustness of the synthesized results. | Not applicable; no sensitivity analysis was conducted. | Not applicable |
| Reporting biases | 21 | Present assessments of risk of bias due to missing results (arising from reporting biases) for each synthesis assessed. | No formal assessment of reporting bias due to missing results was performed. | Not assessed |
| Certainty of evidence | 22 | Present assessments of certainty (or confidence) in the body of evidence for each outcome assessed. | No formal certainty-of-evidence assessment was performed. | Not assessed |
| DISCUSSION | | | | |
| Discussion | 23a | Provide a general interpretation of the results in the context of other evidence. | Pages 12-13, Section 4 | Reported |
| Discussion | 23b | Discuss any limitations of the evidence included in the review. | Page 14, Section 6 | Reported |
| Discussion | 23c | Discuss any limitations of the review processes used. | Page 14, Section 6 | Reported |
| Discussion | 23d | Discuss implications of the results for practice, policy, and future research. | Page 11, Section 3.8; page 13, Section 5; page 15, Section 7 | Reported |
| OTHER INFORMATION | | | | |
| Registration and protocol | 24a | Provide registration information for the review, including register name and registration number, or state that the review was not registered. | Page 5, Section 2.1; PROSPERO CRD420261433767 | Reported |
| Registration and protocol | 24b | Indicate where the review protocol can be accessed, or state that a protocol was not prepared. | Page 5, Section 2.1; PROSPERO CRD420261433767 | Reported |
| Registration and protocol | 24c | Describe and explain any amendments to information provided at registration or in the protocol. | No protocol amendments are described in the manuscript. | Not reported |
| Support | 25 | Describe sources of financial or non-financial support for the review, and the role of the funders or sponsors in the review. | Page 15, Funding and Acknowledgements | Reported |
| Competing interests | 26 | Declare any competing interests of review authors. | Page 15, Competing interests | Reported |
| Availability of data, code and other materials | 27 | Report on which materials are publicly available and where they can be found: template data collection forms; data extracted from included studies; data used for analyses; analytic code; and other review materials. | Page 15, Data availability statement; Supplementary file 2; Tables 1-3 | Partially reported |
